# The first report of the mobile colistin resistance gene, *mcr-10.1*, in Kenya and a novel mutation in the *phoQ* gene (S244T) in a colistin-resistant *Enterobacter cloacae* clinical isolate

**DOI:** 10.1128/spectrum.01855-23

**Published:** 2024-01-17

**Authors:** Collins Kigen, Angela Muraya, James Wachira, Lillian Musila

**Affiliations:** 1 Department of Emerging Infectious Diseases, USAMRD—Africa, Kenya, Nairobi, Kenya; 2 Department of Emerging Infectious Diseases, Kenya Medical Research Institute, Nairobi, Kenya; 3 Department of Biochemistry, Jomo Kenyatta University of Agriculture and Technology, Nairobi, Kenya; Emory University School of Medicine, Atlanta, Georgia, USA

**Keywords:** antimicrobial resistance, colistin, *mcr-10*, Kenya

## Abstract

This study describes the identification of the *mcr-10.1* gene in a clinical isolate of an ST1 *Enterobacter cloacae* isolate cultured in 2015 in Kenya. The isolate was multidrug resistant, phenotypically non-susceptible to various antibiotics, including colistin. Whole genome sequence analyses indicated carriage of chromosomally encoded antimicrobial resistance genes and the colistin-resistant gene *mcr-10.1* located on a 72-kb plasmid designated pECC011b with an IncFIA(HI1) replicon directly adjacent to tyrosine recombinase gene, *xerC*, and downstream of an IS*KPn26* insertion sequence. Studies have shown that expression of *mcr-10.1* may not be sufficient to confer colistin resistance, but a novel non-synonymous mutation (S244T) was identified in the *phoQ* gene known to influence colistin resistance within lipid modification pathways, which could have complemented the *mcr-10.1* resistance mechanism. *In silico* analysis of the mutant *phoQ* protein shows the location of the mutation to be at the Histidine kinases, Adenyl cyclases, Methyl-accepting proteins and Phosphatases (HAMP) region, which plays a crucial role in the protein’s activity. This study and our previous report of *mcr-8* in *Klebsiella pneumoniae* indicate the presence of mobile *mcr* genes in the *Enterobacterales* order of bacteria in Kenya. The study points to the importance of regulation of colistin in the animal industry and enhancing surveillance in both human and animal health to curb the spread of *mcr* genes and accurately assess the risks posed by these mobile genetic elements in both sectors.

**IMPORTANCE:**

This paper reports the detection of new colistin resistance mechanisms in Kenya in a clinical isolate of *Enterobacter cloacae* in a patient with a healthcare-associated infection. The plasmid-mediated resistance gene, *mcr-10.1*, and a novel amino acid mutation S244T in the *phoQ* gene, located in a region of the protein involved in membrane cationic stability contributing to colistin resistance, were detected. Colistin is a critical last-line drug for multidrug-resistant (MDR) gram-negative human infections and is used for treatment and growth promotion in the animal industry. The emergence of the resistance mechanisms points to the potential overuse of colistin in the animal sector in Kenya, which enhances resistance, threatens the utility of colistin, and limits treatment options for MDR infections. This study highlights the need to enhance surveillance of colistin resistance across sectors and strengthen One Health policies that ensure antimicrobial stewardship and implementation of strategies to mitigate the spread of antibiotic resistance.

## OBSERVATION

Colistin (polymyxin E) is a last-line antibiotic crucial for treating multidrug-resistant (MDR) gram-negative bacterial infections and for treatment and growth promotion in the animal industry (1). The resistance mechanisms include efflux pumps, encapsulation, chromosomal mutations of lipid modification pathway genes, and the plasmid-borne *mcr* genes 10 variants (*mcr* 1 to 10) of these highly mobile genes have been detected in diverse sources globally, which could lead to the loss of this critical last-line drug (2, 3).

In 2015, a colistin-resistant *Enterobacter cloacae* isolate was detected in a 23-year-old female inpatient at a hospital in Nairobi, Kenya, with a healthcare-associated leg wound infection enrolled in an antimicrobial resistance surveillance study (KEMRI2767/WRAIR 2089). The bacterial isolate was identified using the GN-ID card and antimicrobial susceptibility test using the XN05 card on a VITEK2 (Biomerieux, Craponne, France). Colistin resistance was confirmed using a cation-adjusted Mueller Hinton broth dilution assay, including a *Pseudomonas aeruginosa* (ATCC 29853) negative control and a *Proteus mirabilis* positive control. Antibiotic resistance was interpreted according to the Clinical and Laboratory Standards Institute 2022 breakpoints (M100, 32nd edition) (4). Total DNA was extracted using the Quick-DNA Fungal/Bacterial Miniprep Kit (Zymo Research, California, USA). Nanopore sequencing libraries were prepared using the Ligation Sequencing Kit (LSK109) and loaded onto an R10.3 flow cell. Illumina sequencing libraries were prepared using the KAPA HyperPlus Kit (Roche Diagnostics, Indianapolis, IN, USA), quantified using the KAPA Library Quantification Kit–Illumina/Bio-Rad iCycler (Roche Diagnostics, Indianapolis, IN, USA), and sequenced with the MiSeq Reagent Kit v3 (600 cycles) on an Illumina MiSeq (Illumina Inc., San Diego, CA, USA).

The hybrid assembly of the long and short reads was generated using Unicycler v0.5.0 normal mode (5). Contigs were visualized using Bandage v0.8.1 (6), and a BLAST search identified each contig as chromosomal or plasmid. The multilocus sequence type was determined using mlst (https://github.com/tseemann/mlst). Antimicrobial resistance (AMR) genes were detected through the ABRicate v1.0.1 pipeline (https://github.com/tseemann/abricate), querying databases ResFinder (7), CARD (8), and NCBI AMRFinderPlus (9). The hAMRonization tool (https://github.com/pha4ge/hAMRonization) selected unique genes from these databases. Plasmid replicons were identified with PlasmidFinder (10). The plasmid sequence, extracted using Bandage v0.8.1, was annotated with Prokka v1.14.6 (11), and gene features were visualized using SnapGene.

The short reads were trimmed using Trimmomatic v0.39 (12) [to filter off low-quality reads (*Q* score <20) and reads less than 75 bp]. The high-quality reads were then mapped against the reference *Enterobacter cloacae* ATCC 13047 genome (NC_014121.1) using bbmap v39.01 (13). The mapped reads were sorted and indexed with samtools v1.9 (14). Bcftools v1.9 (14) was used to generate genotype likelihood at each genomic position with coverage, followed by calling the variants using the default calling method with a minimum depth of 10. Low-quality variants (QUAL <40) were then filtered out. The filtered variants were annotated against a custom database built using the *Enterobacter cloacae* ATCC 13047 genome (NC_014121.1) with Snpeff v5.0 (15). Finally, the variants of *pmrA/B*, *phoP/Q*, and *mgrB* genes were identified in the output vcf files and visualized using IGV v2.16.0 (16).

The coding nucleotide sequences of wild type and mutant *phoQ* were translated using Expasy’s Translate tool (17). Their 3D structures were predicted with Alphafold2-multimer (18) and MARTINI coarse-grained molecular dynamics simulations were performed as described by Mahmood et al. (19) with slight modifications on the lipid bilayer composition to POPE:POPG (3:1). Visualizations were performed with Molviewer (20).

The ST1 *Enterobacter cloacae* complex isolate exhibited non-susceptibility to amoxicillin-clavulanic acid, cefoxitin, colistin, chloramphenicol, and ampicillin-sulbactam. Colistin resistance was confirmed by the gold standard broth microdilution assay (MIC ≥ 4 µg/mL).

The hybrid assembly yielded a genome of 5,247,180 bp with five contigs, i.e., one chromosome of 5,065,027 bp, three plasmids of 99,848 bp, 72,193 bp, and 4,096 bp, and one unplaced contig of 6,016 bp. The GC content was 54.75% with a mean N50 of 5,065,027 (NCBI genome accession JARXZG000000000). The chromosomally encoded AMR genes confer resistance to antibiotics of the beta-lactams, fluoroquinolone, polypeptide, and fosfomycin classes and MDR efflux pumps (Table 1). The only plasmid-encoded gene was the *mcr-10.1* colistin resistance gene.

**TABLE 1:** Detection of AMR genes, genomic location, and corresponding antibiotic class in the *E. cloacae* clinical isolate

AMR genes	Genome location	Antibiotic class
*bla*_CMH-1_, *bla*_CMH-3_, *bla*_CMH-4_	Chromosome	Beta-lactam
*mcr-10.1*	Plasmid	Polymyxin
*emrB*, *oqxA*, *oqxA10*, *oqxB*, *oqxB9*	Chromosome	Fluoroquinolone
*bacA*	Chromosome	Polypeptide
*fosA*, *fosA2*	Chromosome	Fosfomycin
*mdtB*, *mdtC*, *msbA*, *acrA*, *acrB*, *tolC*, *acrD*, *KpnG*	Chromosome	MDR efflux pumps

**Fig 1:**
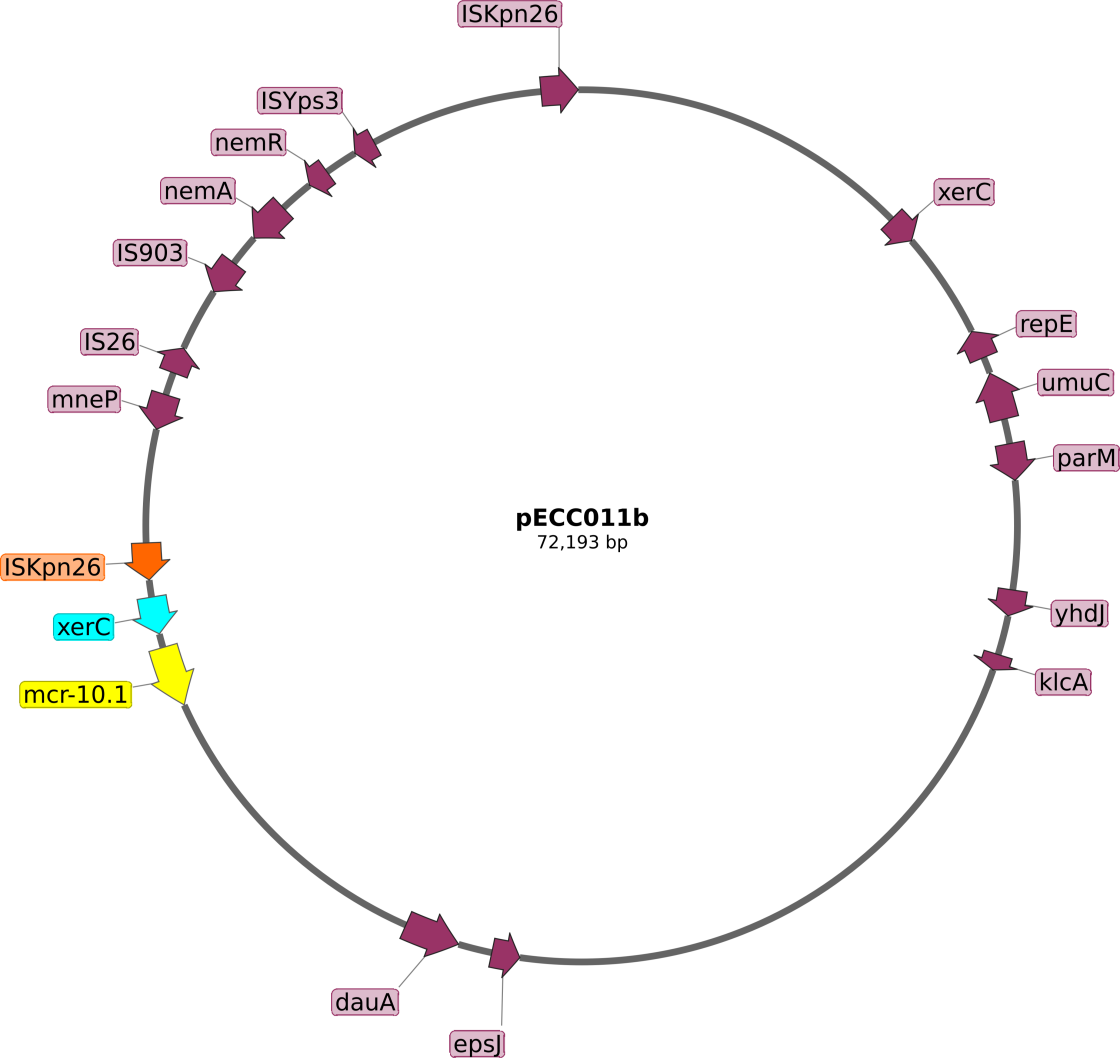
The genetic organization of the plasmid pECC011b (NCBI accession JARXZG010000003) harboring the *mcr-10.1* gene (highlighted in yellow) located downstream of ISKpn26 (highlighted in orange), and XerC (highlighted in cyan). The arrows indicate the direction of translation of the genes, and the circle represents the plasmid’s size, 72,193 bp. Hypothetical proteins were hidden from the map. (The plasmid was annotated with Prokka and visualized with Snapgene.)

The *mcr-10.1* gene was on a plasmid designated pECC011b (Fig. 1). The plasmid had an IncFIA(HI1) replicon and was 99.5% identical to the pECC59-2 (CP080472.1) isolated from a clinical *Enterobacter hormaechei* in China. The *mcr-10.1* gene was adjacent to *xerC*, a site-specific tyrosine recombinase downstream of an IS*KPn26* insertion sequence similar to previous reports of the genomic environment of *mcr-10* (21, 22).

**Fig 2:**
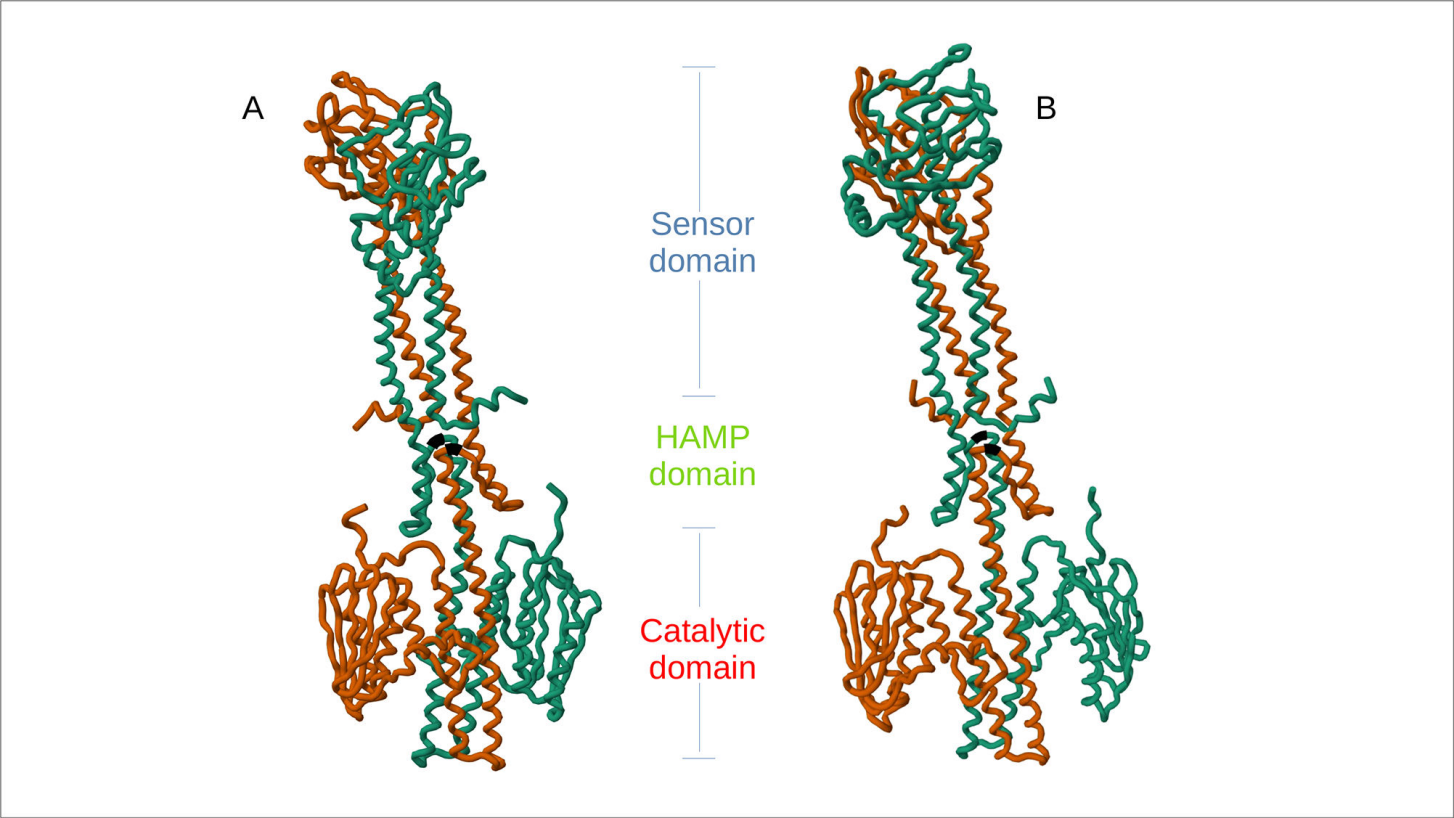
3D snapshots of the wild type *phoQ* (**A**) and mutant *phoQ* (**B**) obtained from trajectory files during simulation displaying the sensor domain, HAMP, and the catalytic domain. The protein is a homodimer (chain 1 in sea blue and chain 2 in brown). The amino acid residue at position 244 (highlighted in black) is serine in the wild type and threonine in the mutated protein.

Colistin resistance can also result from mutations in lipid modification pathway genes, including *phoQ*, *phoP*, *pmrA*, *pmrB*, *and mgrB* (2). A non-synonymous mutation (730T>A) in *phoQ*, leading to serine-to-threonine (S244T) change, was identified. A synonymous mutation (12G>A, L4L) was found in the *phoP* gene, while *pmrA*, *pmrB*, and *mgrB* genes had no mutations. To identify the location of the non-synonymous mutation, top-ranked structures for both wild type and mutant homodimer proteins were selected using Alphafold’s ranking scheme. The predicted 3D structures of wild type and mutant *phoQ* exhibited a consistent arrangement, featuring a periplasmic sensor domain connected to a transmembrane domain, the Histidine kinases, Adenyl cyclases, Methyl-accepting proteins and Phosphatases (HAMP) domain where the S244T mutation was located, and the histidine kinase region. This structural consistency was maintained when the proteins were inserted into a lipid bilayer, as shown in snapshots during simulation (Fig. 2).

The *mcr-10* gene was first described in China in an *Enterobacter roggenkampii* clinical strain (22). Since then, it has been identified in 18 countries on five continents, including Africa, and 15 species of seven genera in the *Enterobacterales* family (23, 24). This study identified an *E. cloacae* isolate with an *mcr-10.1* gene, the first report in Kenya and the only one globally in an ST1 isolate. The ability of the *mcr-10-*bearing plasmids to self-transmit, mobilize, and re-integrate indicates that the *mcr-10.1* gene has a high potential to spread within the *Enterobacterales* family in Kenya. Future conjugation experiments would confirm the transmissibility of the *mcr-10.1* gene in this context. The *phoQ* gene mutation was novel, so its potential impact on protein function was assessed *in silico*. Molecular dynamic simulations revealed that the S244T mutation was in the HAMP region. As demonstrated in a study by Mensa et al. (25), mutations in this region can disrupt the balance between the sensor and catalytic domains, potentially increasing kinase activity and causing cationic imbalance, ultimately affecting colistin susceptibility (25).

In summary, this study presents emerging mechanisms of colistin resistance in an *E. cloacae* isolate in Kenya, with the first report of the mobile *mcr-10.1* gene, which fills gaps in mapping global antimicrobial resistance. Although this was detected in a single isolate, the mobility of the associated plasmid indicates a high risk of colistin resistance transmission within and between species. As colistin is considered a last-resort antibiotic for treating gram-negative bacterial infections and is used extensively in animal production, robust policies are needed to enhance antimicrobial stewardship to curb *mcr* gene spread and preserve its efficacy.

## Data Availability

The whole genome sequence of the *Enterobacter cloacae* isolate has been deposited at DDBJ/ENA/GenBank under the accession no. JARXZG000000000. The raw read sequences have been deposited in the NCBI SRA database under the accession no. SRR24415236 (Illumina sequencing reads) and SRR24415235 (Nanopore sequencing reads).

## References

[1] Sharma J, Sharma D, Singh A, Sunita K, Donadu MG. 2022. Colistin resistance and management of drug resistant infections. Can J Infect Dis Med Microbiol 2022:4315030. doi:10.1155/2022/4315030 36536900 PMC9759378

[2] Aghapour Z, Gholizadeh P, Ganbarov K, Bialvaei AZ, Mahmood SS, Tanomand A, Yousefi M, Asgharzadeh M, Yousefi B, Kafil HS. 2019. Molecular mechanisms related to colistin resistance in Enterobacteriaceae. Infect Drug Resist 12:965–975. doi:10.2147/IDR.S199844 31190901 PMC6519339

[3] Bastidas-Caldes C, de JH, Salgado MS, Villacís MJ, Coral-Almeida M, Yamamoto Y, et al.. 2022. Worldwide prevalence of mcr-mediated colistin-resistance Escherichia coli in Isolates of clinical samples, healthy humans, and livestock—a systematic review and meta-analysis. Pathogens 11:659. 35745513 10.3390/pathogens11060659 PMC9230117

[4] Clinical and laboratory standards Institute. M100. 2022. In Performance standards for antimicrobial susceptibility testing, 32nd edition

[5] Wick RR, Judd LM, Gorrie CL, Holt KE. 2017. Unicycler: resolving bacterial genome assemblies from short and long sequencing reads. PLoS Comput Biol 13:e1005595. doi:10.1371/journal.pcbi.1005595 28594827 PMC5481147

[6] Wick RR, Schultz MB, Zobel J, Holt KE. 2015. Bandage: interactive visualization of de novo genome assemblies. Bioinformatics 31:3350–3352. doi:10.1093/bioinformatics/btv383 26099265 PMC4595904

[7] Bortolaia V, Kaas RS, Ruppe E, Roberts MC, Schwarz S, Cattoir V, Philippon A, Allesoe RL, Rebelo AR, Florensa AF, et al.. 2020. Resfinder 4.0 for predictions of phenotypes from genotypes. J Antimicrob Chemother 75:3491–3500. doi:10.1093/jac/dkaa345 32780112 PMC7662176

[8] Alcock BP, Raphenya AR, Lau TTY, Tsang KK, Bouchard M, Edalatmand A, Huynh W, Nguyen A-L, Cheng AA, Liu S, et al.. 2020. CARD 2020: antibiotic resistome surveillance with the comprehensive antibiotic resistance database. Nucleic Acids Res 48:D517–D525. doi:10.1093/nar/gkz935 31665441 PMC7145624

[9] Feldgarden M, Brover V, Haft DH, Prasad AB, Slotta DJ, Tolstoy I, Tyson GH, Zhao S, Hsu C-H, McDermott PF, Tadesse DA, Morales C, Simmons M, Tillman G, Wasilenko J, Folster JP, Klimke W. 2019. Validating the AMRFinder tool and resistance gene database by using antimicrobial resistance genotype-phenotype correlations in a collection of isolates. Antimicrob Agents Chemother 63:e00483-19. doi:10.1128/AAC.00483-19 31427293 PMC6811410

[10] Carattoli A, Zankari E, García-Fernández A, Voldby Larsen M, Lund O, Villa L, Møller Aarestrup F, Hasman H. 2014. In Silico detection and typing of plasmids using PlasmidFinder and plasmid multilocus sequence typing. Antimicrob Agents Chemother 58:3895–3903. doi:10.1128/AAC.02412-14 24777092 PMC4068535

[11] Seemann T. 2014. Prokka: rapid prokaryotic genome annotation. Bioinformatics 30:2068–2069. doi:10.1093/bioinformatics/btu153 24642063

[12] Bolger AM, Lohse M, Usadel B. 2014. Trimmomatic: a flexible trimmer for illumina sequence data. Bioinformatics 30:2114–2120. doi:10.1093/bioinformatics/btu170 24695404 PMC4103590

[13] Bushnell B. 2014. BBMap: a fast, accurate, splice-aware aligner. Available from: https://escholarship.org/uc/item/1h3515gn

[14] Danecek P, Bonfield JK, Liddle J, Marshall J, Ohan V, Pollard MO, Whitwham A, Keane T, McCarthy SA, Davies RM, Li H. 2021. Twelve years of SAMtools and BCFtools. Gigascience 10:giab008. doi:10.1093/gigascience/giab008 33590861 PMC7931819

[15] Cingolani P, Platts A, Wang LL, Coon M, Nguyen T, Wang L, Land SJ, Lu X, Ruden DM. 2012. A program for annotating and predicting the effects of single nucleotide polymorphisms, SnpEff: SNPs in the genome of Drosophila melanogaster strain w1118; iso-2; iso-3. Fly (Austin) 6:80–92. doi:10.4161/fly.19695 22728672 PMC3679285

[16] Robinson JT, Thorvaldsdóttir H, Winckler W, Guttman M, Lander ES, Getz G, Mesirov JP. 2011. Integrative genomics viewer. Nat Biotechnol 29:24–26. doi:10.1038/nbt.1754 21221095 PMC3346182

[17] Artimo P, Jonnalagedda M, Arnold K, Baratin D, Csardi G, de Castro E, Duvaud S, Flegel V, Fortier A, Gasteiger E, Grosdidier A, Hernandez C, Ioannidis V, Kuznetsov D, Liechti R, Moretti S, Mostaguir K, Redaschi N, Rossier G, Xenarios I, Stockinger H. 2012. ExPASy: SIB bioinformatics resource portal. Nucleic Acids Res 40:W597–603. doi:10.1093/nar/gks400 22661580 PMC3394269

[18] Mirdita M, Schütze K, Moriwaki Y, Heo L, Ovchinnikov S, Steinegger M. 2022. ColabFold: making protein folding accessible to all. Nat Methods 19:679–682. doi:10.1038/s41592-022-01488-1 35637307 PMC9184281

[19] Mahmood MI, Poma AB, Okazaki K-I. 2021. Optimizing Gō-MARTINI coarse-grained model for F-BAR protein on lipid membrane. Front Mol Biosci 8:619381. doi:10.3389/fmolb.2021.619381 33693028 PMC7937874

[20] Sehnal D, Bittrich S, Deshpande M, Svobodová R, Berka K, Bazgier V, Velankar S, Burley SK, Koča J, Rose AS. 2021. Mol* viewer: modern web app for 3D visualization and analysis of large biomolecular structures. Nucleic Acids Res 49:W431–W437. doi:10.1093/nar/gkab314 33956157 PMC8262734

[21] Liao W, Cui Y, Quan J, Zhao D, Han X, Shi Q, Wang Q, Jiang Y, Du X, Li X, Yu Y. 2022. High prevalence of colistin resistance and mcr-9/10 genes in Enterobacter spp. in a tertiary hospital over a decade. Int J Antimicrob Agents 59:106573. doi:10.1016/j.ijantimicag.2022.106573 35307563

[22] Wang C, Feng Y, Liu L, Wei L, Kang M, Zong Z. 2020. Identification of novel mobile colistin resistance gene mcr-10. Emerg Microbes Infect 9:508–516. doi:10.1080/22221751.2020.1732231 32116151 PMC7067168

[23] Xu L, Wan F, Fu H, Tang B, Ruan Z, Xiao Y, et al.. n.d. Emergence of colistin resistance gene *mCR - 10* in *Enterobacterales* isolates recovered from fecal samples of chickens, slaughterhouse workers, and a nearby resident, p e00418–22. In Khursigara CM (ed), Microbiol Spectr. 2022 Apr. Vol. 10. 10.1128/spectrum.00418-22 PMC9045214 35412362

[24] Guan J, Li L, Zheng L, Lu G, Wang Y, Lakoh S, Sevalie S, Jiang B, Ji X, Sun Y, Liu J, Zhu L, Guo X, Tyne DV. 2022. First report of the colistin resistance gene mcr-10.1 carried by Inc_pA1763-KPC_ plasmid pSL12517-mcr10.1 in Enterobacter cloacae in Sierra Leone. Microbiol Spectr 10:e0112722. doi:10.1128/spectrum.01127-22 35695522 PMC9431528

[25] Mensa B, Polizzi NF, Molnar KS, Natale AM, Lemmin T, DeGrado WF. 2021. Allosteric mechanism of signal transduction in the two-component system histidine kinase phoQ. Elife 10:e73336. doi:10.7554/eLife.73336 34904568 PMC8719878

